# Influences of Smoking in Traditional and Industrial Conditions on Flavour Profile of Harbin Red Sausages by Comprehensive Two-Dimensional Gas Chromatography Mass Spectrometry

**DOI:** 10.3390/foods10061180

**Published:** 2021-05-24

**Authors:** Xiaoyu Yin, Qian Chen, Qian Liu, Yan Wang, Baohua Kong

**Affiliations:** 1 College of Food Science, Northeast Agricultural University, Harbin 150030, China; yinxiaoyu@neau.edu.cn (X.Y.); chenqian@neau.edu.cn (Q.C.); liuqian@neau.edu.cn (Q.L.); 2 Shimadzu (China) Co., Ltd., Shenyang 110000, China; shewyan@shimadzu.com.cn

**Keywords:** Harbin red sausages, smoking methods, quality characteristics, volatile compounds

## Abstract

Smoking is mainly used to impart desirable flavour, colour and texture to the products. Various food smoking methods can be divided into traditional and industrial methods. The influences of three different smoking methods, including traditional smouldering smoke (TSS), industrial smouldering smoke (ISS) and industrial liquid smoke (ILS), on quality characteristics, sensory attributes and flavour profiles of Harbin red sausages were studied. The smoking methods had significant effects on the moisture content (55.74–61.72 g/100 g), L*-value (53.85–57.61), a*-value (11.97–13.15), b*-value (12.19–12.92), hardness (24.25–29.17 N) and chewiness (13.42–17.32). A total of 86 volatile compounds were identified by headspace solid phase microextraction combined with comprehensive two-dimensional gas chromatography mass spectrometry (GC × GC-qMS). Among them, phenolic compounds were the most abundant compounds in the all sausages. Compared with sausages smoked with smouldering smoke, the ILS sausages showed the highest content of volatile compounds, especially phenols, alcohols, aldehydes and ketones. Principal component analysis showed that the sausages smoked with different methods had a good separation based on the quality characteristics and GC × GC-qMS data. These results will facilitate optimising the smoking methods in the industrial production of smoked meat products.

## 1. Introduction

Smoking as a method of food preservation helps prolong product shelf life [1]. Smoking is mainly used to impart desirable flavour, colour and texture to the products [2]. Various food smoking methods can be divided into traditional and industrial methods. Additionally, the smoke generation technologies can be classified as liquid smoke, friction smoke, steam smoke, fluidisation smoke, electrostatic smoke, touch smoke, and smouldering smoke [3]. Generally, smoke generation is most commonly achieved with smouldering smoke at temperatures between 500 and 800 °C, controllable by air supply [2]. At present, the most frequently used smoking methods are traditional smouldering smoke and industrial smouldering smoke in the processing of smoked meat products. Traditional smouldering smoke is developed by smouldering wood directly below hanging meat in a smokehouse for a long time [3]. The temperature, humidity and density of smoke must be skilfully controlled by the operator by changing the moistness of the wood chips or sawdust or opening or closing the air inlets of the smokehouse [4].

In industrial production of smoked meat products, the smoking procedure is generally carried out in an automatic smokehouse. In industrial smouldering-type generators, wood chips or sawdust are automatically fed onto a grated fire bed or electrically heated plate, which produce smoke more efficiently under controlled conditions. Furthermore, char particles can settle during the passage of smoke through the system of pipes and minimise the generation of various unhealthy compounds of meat products, especially polycyclic aromatic hydrocarbons (PAHs) [2]. Liquid smoke is a more modern method and is produced by condensing wood smoke formed by the controlled, minimal oxygen pyrolysis of sawdust or wood chips. Liquid smoke is considered a healthier smoking method, reduces the processing time and weight loss of traditional smouldering smoking, and eliminates nitrogen oxides and PAH levels in smoked meat products, giving much of the desired colour and flavour of conventional smoking, thereby creating new technological possibilities [5].

As a typical smoked meat product, Harbin red sausage is highly appreciated by consumers in northern China due to its sensory attributes, especially its unique flavour [6]. During the thermal processing of Harbin red sausage, many biochemical reactions (e.g., lipolysis, proteolysis, oxidation and Maillard reactions) take place and contribute to flavour development [7]. Additionally, the smoke components and their interactions with the meat play important roles in the overall flavour of Harbin red sausages.

In the flavour research field, sensory evaluation is one of the basic direct methods to describe sensory attributes. However, some studies have indicated that the perceptions of a trained panel do not completely reflect the sensory perceptions of consumers [3,8]. It has become increasingly important to include instrumental data to help understand the sensory attributes of foodstuffs [9]. Recently, advanced multidimensional analytical platforms based on comprehensive two-dimensional gas chromatography mass spectrometry (GC × GC-qMS) have been applied to identify volatile compounds of different meats and meat products, such as fresh and grilled eel, dry-cured hams and braised chicken, due to high resolution, high sensitivity and large peak capacity [8,10,11].

Up to now, available information on Harbin red sausage only refers to microorganisms, quality characteristics and sensory attributes of the product; there is a lack of knowledge on the flavours and preferences of smoked meat products associated with different smoke generation technology. Hence, the objective of this study was to evaluate the influences of different smoking methods on the flavour profile of Harbin red sausage by a combination of headspace solid-phase microextraction (HS-SPME) and GC × GC-qMS. Comparisons of the quality characteristics of Harbin red sausage smoked with different methods were also conducted.

## 2. Materials and Methods

### 2.1. Sausage Preparation

Three independent batches of sausages (replicates) were prepared, and a total of three groups of sausages were prepared in each batch: sausages smoked with traditional smouldering smoke (TSS), industrial smouldering smoke (ISS) and industrial liquid smoke (ILS). Lean pork and pork backfat were purchased from Dazhuangyuan Industrial Co. (Harbin, China). The Harbin red sausages were manufactured according to the method of Lv et al. [6] with some modifications. For each group of sausages, the sausages were prepared with a basic formula of lean pork 750 g, pork back fat 190 g, starch 60 g, salt 28.2 g, sodium nitrite 0.075 g, sodium erythorbate 0.375 g, alkaline phosphate 2.25 g, monosodium glutamate 2.0 g, ground pepper 2.0 g, garlic 5.0 g and ice water 250 g. Lean pork and back fat, previously minced using a grinder (HYTW-32C, 3.7 kW/380 V, HOYING, Ningbo, Zhejiang, China) with a 4-mm plate, were cured with a curing agent (salt, sodium erythorbate, sodium nitrite and alkaline phosphate) and salted (salt) at 4 °C for 20 h. Thereafter, lean pork and back fat were mixed with ice water, starch and spices in a vacuum mixer machine (BX150, Hengshun Machinery Factory, Jinan, Shandong, China). For ILS sausages, 3.5 g liquid smoke (Red Arrow) was also added. Then, the pork batter was filled into 38-mm diameter porcine natural casings with a sausage stuffer (SW-3, Six Electrical Machinery Co., Ltd., Guangzhou, Guangdong, China). The raw sausages were roasted at 70 °C for 40 min followed by cooking at 85 °C for approximately 30 min until internal temperature reached 74 °C. The TSS sausages were smoked for 12 h in a smokehouse. The sausages, at 2.5 m from smouldering wood, were hung onto shelves placed above a hearth. The smokehouse was maintained at a temperature between 49.5 and 65.5 °C (average 60 °C). The ISS sausages were smoked for 2 h at 60 °C in an automatic smokehouse (YXQ2-2, 9.5 kW/380 V, XIAOJIN, Shjiazhuang, Hebei, China). Similarly, the ILS sausages were roasted for 2 h at 60 °C in the automatic smokehouse. Finally, the sausages were stored under refrigeration (4 °C) and analysed within one day after sausage preparation.

### 2.2. Moisture Content

Moisture content was calculated by weight loss according to AOAC method 925.04 [12]. Minced samples (3 g each sample) were dried in an oven at 105 °C for 2–4 h. Then they were cooled to room temperature in desiccator and weighed. Drying, cooling and weighing was repeated until the results of two successive weightings did not vary by >0.1% by weight of the sample.

### 2.3. Colour

Colour measurements were performed by a ZE-6000 colourimeter (Nippon Denshoku, Kogyo Co., Tokyo, Japan) using a D 65 light source and a 10° observer with an 8-mm diameter measuring area and a 50-mm diameter illumination area. A white standard plate (L* = 149 95.26, a* = −0.89, b* = 1.18) was used for calibration prior to measurements. Results were obtained from three different parts of the sausages as L*-value (lightness), a*-value (redness) and b*-value (yellowness), and the average value was recorded.

### 2.4. Texture Profile Analysis

Texture profile analysis (TPA) was carried out using a texture analyser (TA-XT2 plus, Stable Micro Systems Ltd., Surrey, UK) fitted with a cylindrical probe (P50, 50 mm diameter). Samples were cut into cylinders (20 mm diameter × 20 mm height) from the central portion of each sausage without casing at room temperature. Each cylindrical sausage core was axially compressed to 40% of their original height and subjected to a two-cycle compression test. According to the following characteristics of hardness (N), springiness, cohesiveness, chewiness (N) and resilience, texture measurements were performed under the following conditions: pretest speed 5.0 mm/s, test speed 2.0 mm/s, post-test speed 2.0 mm/s, distance 8.0 mm and force 5.0 g. Eight cylinders of each group of sausages were prepared for TPA analysis.

### 2.5. Sensory Analysis

The sausages were submitted to sensory evaluation to ascertain whether differences existed between samples with different smoking methods according to the method of Han et al. [13] with some modifications. Sausages were served to 20 qualified panellists trained by following the AMSA [14] guidelines across three sessions. A “warm-up” sample at the beginning of each session was evaluated to acquaint panellists with the scoring system. Sausage slices (2-mm thick) were served randomly on white plastic plates, coded with randomised 3-digit numbers. Appearance (intensity of red), texture (hardness), odour (smoky odour and meaty odour) and taste (saltiness) of sausages were evaluated using a 7-point line scale: for intensity of red, 1 = light pink, 2 = antique pink, 3 = tomato red, 4 = beige red, 5 = brown red, 6 = oxide red, 7 = black red (refer to Pantone colour book 7605CP for colours); for smoky odour, 1 = nondetectable, 2 = very bland, 3 = moderately bland, 4 = slightly bland, 5 = slightly intense, 6 = moderately intense, 7 = very intense; for meaty odour, 1 = nondetectable, 2 = very bland, 3 = moderately bland, 4 = slightly bland, 5 = slightly intense, 6 = moderately intense, 7 = very intense; for saltiness, 1 = nondetectable, 2 = very bland, 3 = moderately bland, 4 = slightly bland, 5 = slightly intense, 6 = moderately intense, 7 = very intense; for hardness, 1 = very tough, 2 = moderately tough, 3 = slightly tough, 4 = just about right, 5 = slightly tough, 6 = moderately tough, 7 = very tender. Panellists marked on the scale (numbered 1–7) for each attribute. The panellists cleansed their mouths/taste buds with water between samples. The evaluations were conducted under normal light at room temperature (approximately 25 °C).

### 2.6. Volatile Compound Analysis

Volatile compounds in Harbin red sausages were extracted by HS-SPME using 50/30 μm thickness of divinylbenzene/carboxen/polydimethylsiloxane (DVB/CAR/PDMS) fibre in a manual SPME needle and holder (ANPEL Laboratory Technologies, Inc., Shanghai, China). Minced sausages (4.00 g) were weighed into a 20-mL headspace vial (CNW Technologies, Duesseldorf, Germany) with internal standard 4.00 μL 1,2-dichlorobenzene (100 mg/L in methanol). Following equilibration at 50 °C for 20 min, the SPME fibre was conditioned prior to heating in a gas chromatograph injection port at 240 °C for 60 min and subsequently exposed to the headspace above the sample for 30 min. The absorbed volatile compounds were identified and quantified by GC × GC-qMS.

A comprehensive two-dimensional gas chromatograph and a QP 2020NX plus mass spectrometer (GC × GC-MS-QP 2020NX, Shimadzu Co., Kyoto, Japan) were used to identify and quantify the volatile compounds in Harbin red sausages. Two columns of different types are connected in series via the ZX1-GC × GC Modulator, which is a unit that enables data sampling for GC × GC. The first-dimension column was a nonpolar analytical column (SH-Rtx-1ms 30 m × 0.25 mm × 0.25 μm, Shimadzu Co., Ltd., Kyoto, Japan), and the second-dimension column was a medium polar analytical column (BPX-50 2.5 m × 0.1 mm × 0.1 μm, SGE Analytical Science, Ringwood, Victoria, Australia). The modulation period was 6 s, and the hot jet width was 350 ms. The initial oven temperature of the GC was maintained at 40 °C for 2 min. Then, the temperature was heated to 230 °C at 3 °C/min and held for 35 min. The split injection inlet had a split ratio of 5:1 at 240 °C. Helium was used as carrier gas at a constant flow rate of 1.2 mL/min. The transfer line into the MS source was heated at 260 °C, and the electron impact ionisation source operated at 230 °C with an ionisation energy of 70 eV. The data acquisition rate was 50 Hz over a mass range of 45–339 amu. Data processing software was GC-image (software for multidimensional chromatography, Zoex Corp., Lincoln, NE, USA). The volatile compounds were identified based on a reverse match factor NIST17 library search (similarity > 800). The content of a volatile compound was calculated by a semiquantitative method, and it was calculated by dividing the peak volume of the compound by the peak volume of the internal standard (1,2-dichlorobenzene) and multiplying this ratio by the initial concentration of the internal standard, which was expressed as μg/kg dry matter (DM).

### 2.7. Statistical Analysis

Three independent batches of sausages (replicates) were conducted on the different days, and a total of three groups of sausages were prepared in each batch. All measurements were carried out in triplicate (triplicate observations) for each batch of sausages. Data analysis was accomplished using the SPSS 20.0 software (SPSS, Inc., Chicago, IL, USA). The differences were analysed using analysis of variance (ANOVA) and Tukey test (*p* < 0.05) and the results were expressed as the mean ± standard error (SE). Principal component analysis (PCA) was conducted based on dimension reduction.

## 3. Results

### 3.1. Moisture Content

The moisture content analysis of Harbin red sausages smoked with different methods is shown in Table 1. The TSS sausage had significantly lower (*p* < 0.05) moisture content compared with the ISS and ILS sausages, which was due to the longer smoking time (12 h) of the TSS sausage, leading to more weight loss. A similar result has been reported by Mastanjević et al. [15] in the smoked sausage, they found that the sausage smoked in a traditional smokehouse had a lower moisture content than that in an automated smoking and ripening chamber. However, there was no significant difference (*p* > 0.05) between the ISS and ILS sausages.

### 3.2. Colour Measurement

Desirable colours that develop in smoked meat products are primarily due to nitrosylmyoglobin formation and coloured smoke components and their interaction with meat [2]. As shown in Table 1, the L*-value was significantly different (*p* < 0.05) among the three groups and was the highest in the ILS sausage, followed by the ISS and TSS sausages, which could be due to differences in thin aqueous layers on muscle surfaces [16]. Additionally, smoking duration had an effect on lightness. Long smoking durations increased the smoke deposition concentration and thus, accelerated their sorption by the meat. In terms of redness, the a*-value of sausages smoked with the traditional method was significantly higher than those of sausages smoked with industrial methods (*p* < 0.05). Traditional smouldering smoking is known to produce a stronger smoke ring and longer smoking durations, which contributes to higher intensity redness in the sausages [17]. Additionally, reactions between nitric oxide and myoglobin on the meat surface could be promoted during smoking, leading to smoke rings with bright red colours [18]. In this study, the closed system of an industrial smokehouse created a lower combustion temperature and limited the production of nitric oxide [17,18], thus leading to a lower a*-value. With respect to yellowness, the TSS sausage had the highest b*-value, followed by the ISS and ILS sausages (*p* < 0.05). In all, sausages smoked under traditional conditions had more intense red colours compared with sausages processed under industrial conditions.

### 3.3. Texture Profile Analysis (TPA)

The texture profiles (hardness, springiness, cohesiveness, chewiness and resilience) of Harbin red sausages smoked by the different methods are given in Table 1. The TSS sausage showed significantly higher hardness than the other sausages smoked with industrial methods (*p* < 0.05), which could be due to differences in moisture contents [19]. The hardness of the ILS sausage was slightly higher than that of the ISS sausage (*p* < 0.05), which is due to the complex crosslinks caused by interactions between phenolic compounds in the liquid smoke and proteins, enhancing the texture profile of sausages [20]. Chewiness results were similar to those for hardness. Generally, chewiness is a secondary parameter that depends on hardness, which can reflect the results with respect to hardness [21]. There were no significant differences (*p* > 0.05) in springiness, cohesiveness and resilience among Harbin red sausages smoked by the different methods.

### 3.4. Sensory Evaluation

**Figure 1:**
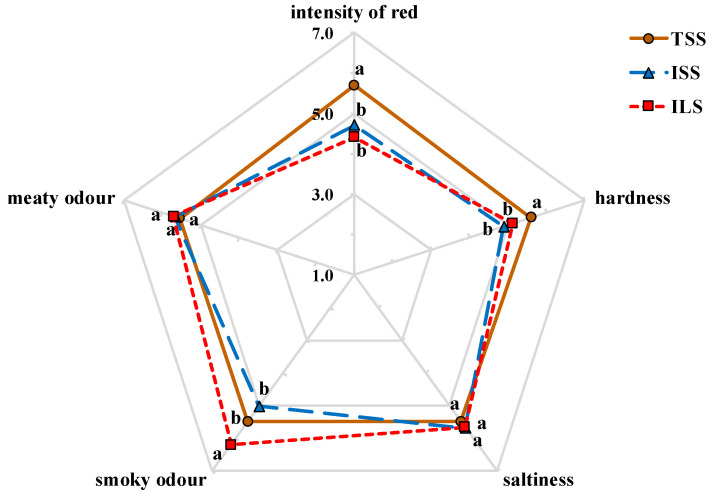
Radar map for sensory analysis of Harbin red sausages smoked in traditional and industrial conditions. TSS: sausages smoked with traditional smouldering smoke; ISS: sausages smoked with industrial smouldering smoke; ILS: sausages smoked with industrial liquid smoke. Different lowercase letters (a, b) mean significant differences among the Harbin red sausages smoked in traditional and industrial conditions (*p* < 0.05).

The smoking process is crucial for the development of the particular sensory attributes of Harbin red sausages, which can affect consumer perception [3]. The appearance, texture, aroma and tastes of Harbin red sausages smoked in traditional and industrial conditions are shown in Figure 1. In appearance, the TSS sausage tended to have a higher red colour intensity than those of the other two groups (*p* < 0.05), which was consistent with the a*-value results. Additionally, the TSS sausage showed a higher score for hardness (*p* < 0.05), which agreed with the texture profile analysis. For odour, the smoky odour score of the ILS sausage was the highest (*p* < 0.05). In terms of the smouldering smoking method, the TSS sausage had a stronger smoky aroma than the ISS sausage, which may be due to the longer smoking duration. No significant differences were observed for meaty odour or saltiness between traditional smoked sausages and industrial smoked sausages (*p* > 0.05).

### 3.5. Volatile Compounds in the Harbin Red Sausages

**Figure 2:**
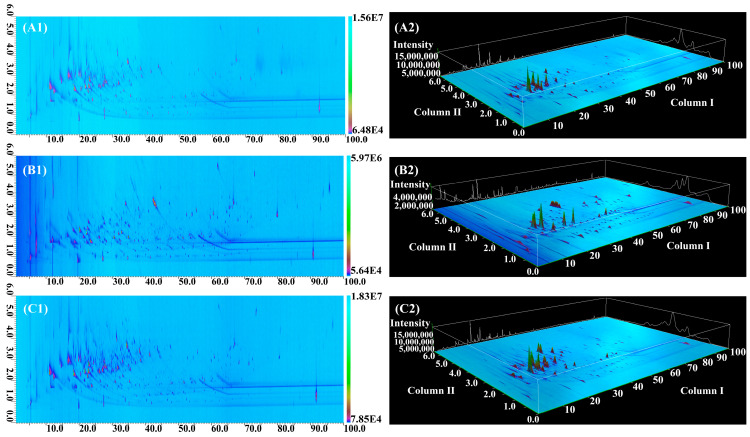
GC × GC-qMS chromatograms of 2D and 3D diagram of volatile compounds from Harbin red sausages with traditional smouldering smoke (**A1**,**A2**), industrial smouldering smoke (**B1**,**B2**) and industrial liquid smoke (**C1**,**C2**). A blob in the 2D image represents one compound, and the bar at the offside of the chromatogram is legend of content of compounds.

**Figure 3:**
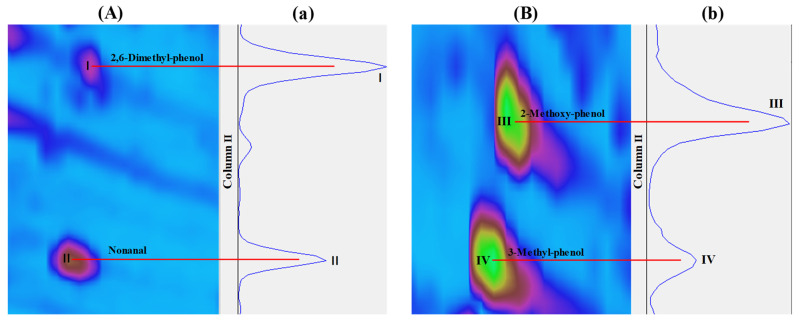
GC × GC plot (the peaks overlapping in the first chromatography column) (**A**,**B**) corresponding to the chromatography in 2nd dimension (**a**,**b**).

To gain a comprehensive understanding of the aroma characteristics of Harbin red sausages, GC × GC-qMS was used to analyse the contents of volatile compounds. Comprehensive 2D and 3D diagrams of the compounds separated by GC × GC are shown in Figure 2. The column I axis is the retention time of the compounds, which is dependent on the carbon number, whereas the column II axis refers to the chemical polarity. As shown in Figure 2, many volatile compounds significantly overlapped in column I due to their similar carbon numbers. These compounds were re-injected into column II by the modulator for better separation. For example, 2,6-dimethyl-phenol (I) and nonanal (II) were separated in the column II; the chromatograms and blobs of 2,6-dimethyl-phenol and nonanal in Figure 3A,a. Similarly, as shown in Figure 3B,b, 2-methoxy-phenol (III) and 3-methyl-phenol (IV) were resolved and identified by GC × GC. These compounds are important contributors to the flavour profile of Harbin red sausages.

Based on the GC × GC, 86 volatile compounds were identified in Harbin red sausages smoked with different methods and are shown in Table 2. Totals of 67, 65 and 61 volatile compounds were identified for the TSS, ISS and ILS sausages, respectively.

#### 3.5.1. Alcohols

A total of 11 alcohols were detected in sausage samples, which are mainly generated from the Strecker degradation reactions of amino acids and the oxidation reactions of lipids in meat products [22]. The sum contents ranged from 769.51 to 4221.80 μg/kg DM, which represented 8.94%, 10.16% and 12.34% in the TSS, ISS and ILS sausages, respectively. The ILS sausage had the highest total content of alcohols, which was due to the fact that the liquid smoke was incorporated into the ILS sausage directly. Among them, 1-octanol, characterised by fatty, sharp aroma notes, had concentrations exceeding its odour threshold (110 μg/kg) [23]. Thus, 1-octanol seemingly significantly contributed to the aroma of Harbin red sausage. 2-Furanmethanol, which is a furan derivative, was also detected in all sausages and the content of 2-furanmethanol was the highest of all alcohols in this study. Yu et al. [24] reported that furans were produced by the interaction between Maillard reaction products and smoke components in smoked bacon.

#### 3.5.2. Aldehydes

Twelve aldehydes were detected in this study, which were produced from lipid oxidisation, Strecker degradation of amino acids and Maillard reactions [25,26]. Generally, the threshold values of aldehyde compounds are relatively low with a relatively rich fat fragrance, which plays an important role in the overall flavour of meat products [27]. In terms of the total contents of aldehydes, the ILS sausage had the highest content (4824.60 μg/kg DM), followed by the TSS sausage (1943.40 μg/kg DM) and ISS sausage (1335.70 μg/kg DM) (*p* < 0.05), which represented 16.58%, 17.64% and 14.10% in the TSS, ISS and ILS sausages, respectively. Among them, a majority of the linear aliphatic aldehydes were detected, such as hexanal, nonanal, undecanal and dodecanal. These aldehydes were also found by Saldaña et al. [28] in smoked bacon with different woods. Short chain aliphatic aldehydes (C2-C10) are usually of importance in the flavour of cooked meat products [29]. As the degradation product of oxidised linoleic acid [10], hexenal was detected in the TSS sausage (80.14 μg/kg DM), ISS (71.06 μg/kg DM) sausage and the ILS sausage (16.67 μg/kg DM), respectively. The high content of hexanal in the TSS sausage could be attributed to the longer smoking time of the TSS sausage. Additionally, 2-furaldehyde and benzaldehyde were detected in all samples. 2-Furaldehyde content was the highest (*p* < 0.05) among aldehydes in the ILS sausage, accounting for 65.46% of aldehydes. 2-Furaldehyde is mainly produced from carbohydrate degradation, which has caramel, sweet, butterscotch, brandy, burnt and sugar aromas [30]. Benzaldehyde is related to bitter almond sensory notes, which is due to decomposition of linoleic acid [29].

#### 3.5.3. Ketones

A total of 10 ketones were found in all sausages, which represented 11.74%, 16.50% and 6.01% in the TSS, ISS and ILS sausages, respectively. In this study, 2-hydroxy-3-methyl-2-cyclopenten-1-one and 2,3-dimethyl-2-cyclopenten-1-one showed relatively higher contents than other ketones in all sausages (*p* < 0.05). These two ketones are typical volatile compounds of wood smoke [31] and they act as flavour enhancers and have an important influence on smoky aroma [32]. Similar results have been reported by Saldaña et al. [28] who found 2-hydroxy-3-methyl-2-cyclopenten-1-one was the most abundant among five ketones detected in the smoked bacons.

#### 3.5.4. Acids

Acids are formed from triglyceride hydrolysis and from aldehyde oxidation [10]. In this study, small amounts of four short-chain fatty acids (C2–C10) and two long-chain fatty acids (C11–C18) were detected, accounting for 1.07%, 3.20% and 2.17% of the total contents in the TSS, ISS and ILS sausages, respectively. Among them, acetic acid was present in the highest amount in all sausages, especially in the ILS sausage (*p* < 0.05). Compared with long-chain fatty acids (tetradecanoic acid and n-hexadecanoic acid), the contribution of short-chain fatty acids to the overall flavour of sausages is comparatively high due to their lower thresholds [10].

#### 3.5.5. Esters

Esters are derived from the esterification of alcohols and carboxylic acids, imparting sweet and typical fruity odours [33]. In this study, only two esters were detected, hexadecanoic acid methyl ester and methyl 2-furoate. The highest content of methyl 2-furoate (665.62 μg/kg DM) was detected in the ILS sausage (*p* < 0.05). The total contents of esters in all samples were detected at low amounts (0.57%, 1.01% and 2.50%), which may be related to the loss of esters by volatilisation or hydrolysis during roasting, cooking and smoking [34]. This result was consistent with Domínguez [35] who found that cooking decreased the relative contents of this chemical family.

#### 3.5.6. Phenols

A total of 9 methoxyphenols and 14 phenols were identified, which are powerful aromatic compounds with musty, pungent, acid, smoky, woody, burnt, ashy, cedar, creosote or petroleum-like characteristics [36]. They are mainly responsible for the unique aroma and taste of smoked products [36] due to their low odour threshold values [23]. In this study, phenols were the most abundant group of compounds, which represented 51.24%, 45.21% and 55.89% in the TSS, ISS and ILS sausages, respectively. Compared with the TSS sausage, the ILS sausage contained a higher total content of phenolic compounds (*p* < 0.05), which was probably due to the direct addition of liquid smoke into the ILS sausage in this study. Besides, the TSS sausage showed higher total content of phenols than that in the ISS sausage, which was probably a consequence of the prolonged and more intense smoking process of the TSS sausage [15,37].

#### 3.5.7. Hydrocarbons

A total of 13 hydrocarbons were identified at relatively high contents in TSS (362.52 μg/kg DM), ISS sausage (329.43 μg/kg DM) and ILS sausage (2177.70 μg/kg DM), which represented 3.09%, 4.35% and 6.36% in the TSS, ISS and ILS sausages, respectively. The hydrocarbons are mainly derived from the oxidation and degradation of fatty acids in meat products, which are catalysed by iron in haemoglobin or myoglobin [33]. Most hydrocarbons had marginal impact on overall flavour of Harbin red sausages due to their relatively high odour threshold values [29].

#### 3.5.8. Others

Eleven other compounds were detected that seemed unrelated to the meat flavour. Among them, benzene derivatives benzonitrile, 1,2-dimethoxy-benzene, 1,2,3-trimethoxybenzene and styrene, which are the benzene derivatives, usually are recognised as volatile contaminants in foodstuffs [38]. Indene mainly exists in tar and crude benzene fractions and was not detected in the ILS sausage, but was detected in the TSS sausage and ISS sausage. Naphthalene and 2-methyl-naphthalene, also detected in the TSS sausage and ISS sausage, have been commonly reported in smoked meat products [39]. The higher amounts of indene and naphthalene in the TSS sausage were due to the direct smoking technique, wherein all volatile compounds and char particles reach the sausage surface. In industrial smouldering smoking, char particles settle during the passage of smoke through the system of pipes; the smoke that reaches the sausages is partly purified. Accordingly, the indene and naphthalene levels in the ILS sausage were considerably lower, which may be due to the removal of unwanted tar and PAHs from the liquid smoke through the refining process.

### 3.6. Principal Component Analysis

**Figure 4:**
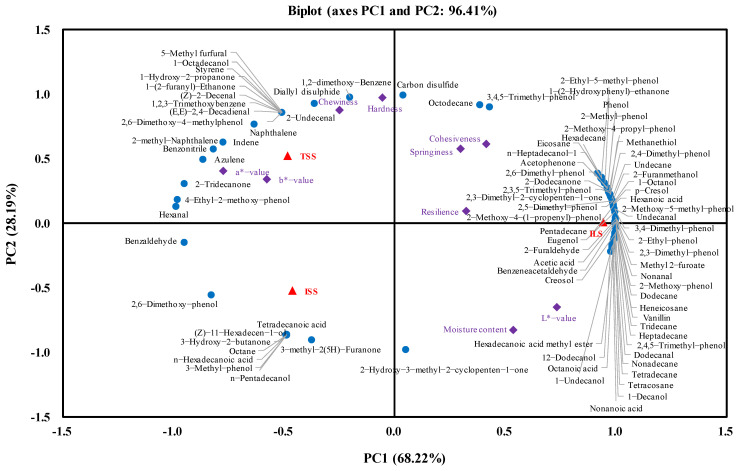
Principal component analysis of the volatile compounds and quality characteristics of Harbin red sausages smoked with different methods. TSS: sausages smoked with traditional smouldering smoke; ISS: sausages smoked with industrial smouldering smoke; ILS: sausages smoked with industrial liquid smoke.

PCA was used to analyse the relationships among physical characteristics and volatile compounds of Harbin red sausages smoked by different methods (Figure 4). The analysis showed that approximately 96.41% of variability was explained by two principal components, indicating that the two principal components covered the vast majority of physical characteristics and the flavour information of the three groups of Harbin red sausages. The first principal component (PC1) was the most important variable in terms of differences among sausages as it accounted for 68.22% of the total variations; the second principal component (PC2) accounted for 28.19% of the total variations. The ILS sausage was located on the positive PC1 axis and positively related to the higher L*-value and the great majority of flavour compounds. PC2 was positively related to hardness and chewiness and was inversely related to the moisture content. The TSS sausage was on the positive PC2 axis, whereas the ISS sausage was on the negative PC2 axis. Therefore, the TSS sausage was characterised by low moisture content and high hardness and chewiness. In summary, PC1 differentiated based on smoke generation method, whereas PC2 distinguished the sausages smoked with traditional method from the sausages smoked with industrial method. 

Regarding the contents of volatile compounds, the smoke generation methods were mainly related to some alcohols (e.g., 2-furanmethanol, 1-octanol, 1-decanol, 1-undecanol and 12-dodecanol), some aldehydes (e.g., 2-furaldehyde, nonanal, undecanal and benzeneacetaldehyde), some acids (e.g., acetic acid, hexanoic acid, octanoic acid and nonanoic acid), most phenols (e.g., p-cresol, 2-methyl-phenol, 2-methoxy-phenol, 2,6-dimethyl-phenol, 2-ethyl-phenol, 2,4-dimethyl-phenol, 2,5-dimethyl-phenol, 2-methoxy-5-methyl-phenol, 2,3-dimethyl-phenol, creosol, 3,4-dimethyl-phenol, 2,4,5-trimethyl-phenol and eugenol) and all hydrocarbons, which were located on the positive PC1 axis. These volatile compounds were more abundant in the ILS sausage. Especially, phenolic compounds are mainly responsible for the unique aroma and taste of smoked products. This may address why the ILS sausage had a more pronounced smoky aroma. Additionally, the traditional smoking method was mainly related to volatile compounds such as 1-octadecanol, (Z)-2-decenal, (E,E)-2,4-decadienal, 2-undecenal, diallyl disulphide, 3,4,5-trimethyl-phenol and 2,6-dimethoxy-4-methylphenol, which were more abundant in the TSS sausage.

## 4. Conclusions

A total of 86 volatile compounds in Harbin red sausages smoked by different smoking methods were identified. Among them, phenols, aldehydes, ketones and alcohols were the dominant volatile compounds contributing to the unique flavour. The ILS sausage showed the highest content of phenolic compounds, leading to a more pronounced smoky aroma. The TSS sausage had higher content of alcohols, aldehydes and phenols than those in the ISS sausage, which was probably a consequence of the prolonged and more intense smoking process of the TSS sausage. Compared with industrial smoking methods, the TSS sausage was characterised by low moisture content and L*-value, and high hardness and chewiness due to a longer smoking time. These results further facilitate the understanding of the flavour profile of Harbin red sausages smoked by different methods and can be used to optimise smoking methods in the industrial production of smoked meat products.

## Figures and Tables

**Table 1:** Moisture content, colour parameters and TPA texture analysis of Harbin red sausages smoked with different methods.

	TSS	ISS	ILS
Moisture content (g/100 g)	55.74 ± 0.06 ^b^	61.50 ± 0.17 ^a^	61.72 ± 0.27 ^a^
L*-value	53.85 ± 0.16 ^c^	56.49 ± 0.06 ^b^	57.61 ± 0.25 ^a^
a*-value	13.15 ± 0.22 ^a^	12.65 ± 0.23 ^b^	11.97 ± 0.17 ^c^
b*-value	12.92 ± 0.25 ^a^	12.61 ± 0.29 ^ab^	12.19 ± 0.20 ^b^
Hardness (N)	29.17 ± 0.56 ^a^	24.25 ± 0.49 ^c^	26.49 ± 0.47 ^b^
Springiness	0.89 ± 0.02 ^a^	0.84 ± 0.03 ^a^	0.89 ± 0.02 ^a^
Cohesiveness	0.78 ± 0.01 ^a^	0.71 ± 0.04 ^a^	0.79 ± 0.02 ^a^
Chewiness (N)	17.32 ± 0.58 ^a^	13.42 ± 0.70 ^b^	14.36 ± 0.71 ^b^
Resilience	0.44 ± 0.03 ^a^	0.44 ± 0.01 ^a^	0.46 ± 0.02 ^a^

^a–c^ Means in the same indexes with different letters differ significantly (*p* < 0.05). TSS: sausages smoked with traditional smouldering smoke; ISS: sausages smoked with industrial smouldering smoke; ILS: sausages smoked with industrial liquid smoke. L*-value: lightness. a*-value: redness. b*-value: yellowness.

**Table 2:** The contents (μg/kg DM) of volatile compounds identified by GC × GC-qMS in Harbin red sausages smoked with different methods.

Compound Name	CAS	Peak I (min)	Peak II (s)	Library Probability	TSS			ISS			ILS		
					Library Match Factor	Library Reverse Match Factor	Content	Library Match Factor	Library Reverse Match Factor	Content	Library Match Factor	Library Reverse Match Factor	Content
**Alcohols**													
2-Furanmethanol	98-00-0	12.30	2.10	55.19	832	842	770.94 ± 15.66 ^b^	825	870	424.88 ± 11.44 ^c^	840	848	2524.80 ± 26.52 ^a^
1-Octanol	111-87-5	22.70	1.72	62.71	750	905	100.23 ± 1.90 ^b^	815	895	53.84 ± 1.86 ^c^	674	870	351.41 ± 8.90 ^a^
1-Decanol	112-30-1	26.00	2.94	9.81	ND	ND	ND	742	839	49.32 ± 0.14 ^b^	687	838	267.53 ± 6.60 ^a^
1-Undecanol	112-42-5	30.50	2.94	12.37	ND	ND	ND	725	813	170.18 ± 2.96 ^b^	724	815	822.41 ± 20.48 ^a^
12-Dodecanol	112-53-8	39.60	2.78	8.16	ND	ND	ND	679	850	31.14 ± 1.08 ^b^	579	824	129.73 ± 2.49 ^a^
n-Pentadecanol	629-76-5	53.70	2.92	13.77	ND	ND	ND	673	852	9.30 ± 0.16	ND	ND	ND
(Z)-11-Hexadecen-1-ol	56683-54-6	54.30	3.02	37.53	ND	ND	ND	600	816	6.21 ± 0.38	ND	ND	ND
n-Heptadecanol-1	1454-85-9	59.80	2.98	28.89	657	848	58.40 ± 2.65 ^b^	718	846	24.62 ± 0.14 ^c^	708	837	125.91 ± 3.27 ^a^
1-Octadecanol	112-92-5	71.60	2.82	26.31	518	806	118.62 ± 3.37	ND	ND	ND	ND	ND	ND
Total							1048.20 ± 23.57 ^b^			769.51 ± 18.15 ^c^			4221.80 ± 68.26 ^a^
**Aldehydes**													
Hexanal	66-25-1	9.80	2.48	67.68	631	814	80.14 ± 2.23 ^a^	584	823	71.06 ± 1.74 ^b^	533	801	16.67 ± 0.81 ^c^
2-Furaldehyde	98-01-1	11.10	2.36	77.48	884	892	984.82 ± 17.65 ^b^	419	893	937.51 ± 28.27 ^b^	903	916	3158.30 ± 38.57 ^a^
Benzaldehyde	100-52-7	16.50	3.90	43.12	614	811	52.98 ± 3.02 ^a^	558	858	58.62 ± 2.01 ^a^	645	812	26.96 ± 2.10 ^b^
5-Methyl furfural	620-02-0	16.70	2.94	75.79	851	879	267.74 ± 15.26	ND	ND	ND	ND	ND	ND
Nonanal	124-19-6	23.80	1.74	93.74	863	896	159.76 ± 2.63 ^b^	900	901	162.31 ± 3.18 ^b^	885	886	639.52 ± 33.46 ^a^
(Z)-2-Decenal	2497-25-8	31.00	1.92	39.03	903	924	115.97 ± 2.74	ND	ND	ND	ND	ND	ND
Undecanal	112-44-7	32.60	2.76	90.62	749	939	34.27 ± 0.11 ^b^	902	955	21.12 ± 0.46 ^c^	844	965	138.69 ± 2.22 ^a^
(E,E)-2,4-Decadienal	25152-84-5	33.30	2.22	33.28	878	898	95.84 ± 1.79	ND	ND	ND	ND	ND	ND
Benzeneacetaldehyde	122-78-1	34.10	4.86	76.21	ND	ND	ND	ND	ND	ND	677	823	187.15 ± 0.74
2-Undecenal	2463-77-6	35.30	1.96	60.03	913	919	111.73 ± 2.05	ND	ND	ND	ND	ND	ND
Vanillin	121-33-5	36.40	3.98	44.37	ND	ND	ND	ND	ND	ND	638	829	141.25 ± 1.25
Dodecanal	112-54-9	51.40	2.90	84.22	697	905	40.13 ± 1.48 ^c^	836	889	85.12 ± 1.27 ^b^	842	888	516.09 ± 14.16 ^a^
Total							1943.40 ± 48.95 ^b^			1335.7 ± 36.93 ^c^			4824.60 ± 93.30 ^a^
**Ketones**													
1-Hydroxy-2-propanone	116-09-6	5.20	2.16	49.94	608	827	187.37 ± 3.17	ND	ND	ND	ND	ND	ND
3-Hydroxy-2-butanone	513-86-0	7.10	2.44	38.71	ND	ND	ND	730	846	314.70 ± 8.70	ND	ND	ND
2-Hydroxy-3-methyl-2-cyclopenten-1-one	765-70-8	19.80	2.96	54.58	828	861	280.86 ± 6.31 ^c^	802	804	536.36 ± 11.11 ^a^	598	812	416.54 ± 9.94 ^b^
2,3-Dimethyl-2-cyclopenten-1-one	80-71-7	20.40	2.96	53.74	802	854	413.96 ± 12.60 ^b^	647	802	273.56 ± 7.98 ^c^	854	867	959.85 ± 25.64 ^a^
Acetophenone	98-86-2	21.80	3.06	72.40	838	904	91.14 ± 1.89 ^b^	ND	ND	ND	794	889	332.92 ± 9.02 ^a^
1-(2-Hydroxyphenyl)-ethanone	118-93-4	26.30	3.00	47.90	675	821	46.23 ± 1.43 ^b^	ND	ND	ND	558	816	150.29 ± 2.26 ^a^
3-methyl-2(5H)-furanone	22122-36-7	34.30	4.16	63.67	543	819	22.37 ± 1.79 ^b^	622	822	77.66 ± 2.06 ^a^	589	807	29.57 ± 2.22 ^b^
1-(2-furanyl)-ethanone	1192-62-7	14.50	2.66	44.65	847	893	248.87 ± 7.60	ND	ND	ND	ND	ND	ND
2-Dodecanone	6175-49-1	47.50	2.88	43.52	871	919	66.92 ± 2.86 ^b^	779	828	34.57 ± 0.20 ^c^	784	843	167.79 ± 2.56 ^a^
2-Tridecanone	593-08-8	48.00	1.88	52.70	702	830	18.37 ± 0.29 ^a^	736	821	12.47 ± 0.08 ^b^	ND	ND	ND
Total							1376.10 ± 37.95 ^b^			1249.30 ± 30.11 ^b^			2056.90 ± 51.64 ^a^
**Acids**													
Acetic acid	64-19-7	5.40	0.94	14.10	876	943	70.47 ± 1.29 ^b^	864	883	72.18 ± 1.23 ^b^	931	934	163.56 ± 7.15 ^a^
Hexanoic acid	142-62-1	18.50	1.72	67.67	636	813	54.77 ± 2.04 ^b^	752	884	32.57 ± 1.37 ^c^	631	843	189.34 ± 3.35 ^a^
Octanoic acid	124-07-2	27.20	2.70	63.77	ND	ND	ND	657	800	32.13 ± 1.74 ^b^	625	823	222.41 ± 5.94 ^a^
Nonanoic acid	112-05-0	31.40	2.74	77.26	ND	ND	ND	759	820	32.49 ± 1.43 ^b^	645	805	166.22 ± 3.12 ^a^
Tetradecanoic acid	544-63-8	49.80	2.94	56.62	ND	ND	ND	777	838	31.71 ± 0.30	ND	ND	ND
n-Hexadecanoic acid	57-10-3	56.20	3.00	65.36	ND	ND	ND	697	862	41.22 ± 0.25	ND	ND	ND
Total							125.24 ± 3.33 ^c^			242.31 ± 6.31 ^b^			741.54 ± 19.56 ^a^
**Esters**													
Methyl 2-furoate	611-13-2	17.40	2.80	48.82	784	801	65.27 ± 1.08 ^b^	746	812	56.83 ± 1.61 ^b^	553	855	665.62 ± 22.98 ^a^
Hexadecanoic acid methyl ester	112-39-0	54.90	2.88	65.55	ND	ND	ND	699	811	19.27 ± 0.06 ^b^	748	843	190.94 ± 1.95 ^a^
Total							66.27 ± 1.08 ^b^			76.10 ± 1.67 ^b^			856.56 ± 24.93 ^a^
**Phenols**													
Phenol	108-95-2	18.20	3.32	56.09	868	898	1684.30 ± 29.68 ^b^	800	869	450.70 ± 11.64 ^c^	865	903	5004.40 ± 69.08 ^a^
2-Methyl-phenol	95-48-7	21.70	2.42	50.68	891	892	424.99 ± 9.61 ^b^	707	853	46.31 ± 0.21 ^c^	890	890	1484.10 ± 39.42 ^a^
3-Methyl-phenol	108-39-4	22.60	3.38	33.06	ND	ND	ND	837	884	29.04 ± 0.35	ND	ND	ND
p-Cresol	106-44-5	22.80	2.44	20.26	897	905	654.25 ± 21.39 ^b^	818	909	233.56 ± 6.20 ^c^	895	903	3099.30 ± 64.05 ^a^
2-Methoxy-phenol	95-05-1	23.00	2.80	69.93	874	907	883.71 ± 15.64 ^b^	817	895	912.57 ± 19.33 ^b^	880	912	2726.40 ± 42.08 ^a^
2,6-Dimethyl-phenol	576-26-1	24.00	2.54	17.65	802	832	104.93 ± 2.14 ^b^	714	821	20.05 ± 0.54 ^c^	790	837	339.89 ± 17.60 ^a^
2-Ethyl-phenol	90-00-6	25.70	2.46	10.06	699	826	84.41 ± 3.18 ^b^	726	821	72.44 ± 3.22 ^b^	791	827	398.59 ± 8.82 ^a^
2,4-Dimethyl-phenol	105-67-9	25.90	3.42	17.31	866	871	247.31 ± 4.99 ^b^	660	814	40.94 ± 1.08 ^c^	863	866	982.11 ± 17.80 ^a^
2,5-Dimethyl-phenol	526-75-0	26.90	3.44	15.58	860	860	193.61 ± 2.45 ^b^	649	822	30.70 ± 0.51 ^c^	859	859	1028.60 ± 30.72 ^a^
2-Methoxy-5-methyl-phenol	1195-09-1	27.30	2.68	50.61	837	865	36.60 ± 0.77 ^b^	811	837	27.04 ± 2.07 ^c^	841	879	102.27 ± 2.23 ^a^
2,3-Dimethyl-phenol	526-75-0	27.50	2.62	30.55	838	869	37.82 ± 0.70 ^b^	715	828	31.87 ± 0.17 ^b^	836	866	203.00 ± 4.03 ^a^
Creosol	93-51-6	27.60	3.68	67.40	882	882	357.18 ± 7.71 ^c^	807	851	468.44 ± 9.39 ^b^	890	890	1416.60 ± 33.20 ^a^
3,4-Dimethyl-phenol	95-65-8	28.20	2.60	27.91	751	813	64.55 ± 1.51 ^b^	ND	ND	31.43 ± 0.77 ^c^	815	839	328.21 ± 8.31 ^a^
2,4,5-Trimethyl-phenol	496-78-6	28.50	2.50	23.22	ND	ND	ND	ND	ND	ND	780	805	194.15 ± 2.93
2-Ethyl-5-methyl-phenol	1687-61-2	29.70	2.46	36.38	808	853	84.48 ± 1.99 ^b^	ND	ND	ND	743	876	242.42 ± 4.78 ^a^
2,3,5-Trimethyl-phenol	697-82-5	29.70	2.68	12.22	659	850	38.39 ± 1.09 ^b^	ND	ND	ND	764	825	163.38 ± 2.20 ^a^
3,4,5-Trimethyl-phenol	527-54-8	31.40	2.58	11.80	798	863	56.55 ± 1.88 ^a^	ND	ND	ND	784	843	52.35 ± 1.30 ^a^
4-Ethyl-2-methoxy-phenol	2785-87-7	31.40	3.58	16.92	860	899	209.60 ± 5.18 ^a^	678	826	167.74 ± 4.12 ^b^	ND	ND	ND
2,6-Dimethoxy-phenol	91-10-1	34.50	3.66	63.67	857	860	355.90 ± 7.80 ^b^	629	813	790.47 ± 11.55 ^a^	ND	ND	ND
Eugenol	97-53-0	35.00	2.66	26.39	797	897	64.32 ± 1.11 ^b^	804	870	40.70 ± 0.66 ^b^	861	908	338.51 ± 15.17 ^a^
2-Methoxy-4-propyl-phenol	2785-87-7	35.60	2.54	95.61	761	848	87.28 ± 1.91 ^b^	ND	ND	ND	812	856	361.57 ± 15.20 ^a^
2-Methoxy-4-(1-propenyl)-phenol	97-54-1	38.80	2.94	69.62	876	926	135.56 ± 3.31 ^b^	805	917	28.83 ± 0.12 ^c^	894	934	658.62 ± 15.22 ^a^
2,6-Dimethoxy-4-methylphenol	6638-05-7	38.40	3.38	50.40	811	859	199.14 ± 3.11	ND	ND	ND	ND	ND	ND
Total							6004.90 ± 127.07 ^b^			3422.80 ± 71.91 ^c^			19124.00 ± 394.15 ^a^
**Hydrocarbons**													
Octane	111-65-9	10.20	3.46	54.21	ND	ND	ND	553	890	64.57 ± 0.28	ND	ND	ND
Undecane	1120-21-4	24.10	2.12	41.88	500	829	50.69 ± 0.99 ^b^	696	898	21.69 ± 0.36 ^c^	476	835	176.72 ± 3.02 ^a^
Dodecane	112-40-3	28.70	2.18	51.94	761	887	16.13 ± 0.30 ^b^	807	900	17.12 ± 0.49 ^b^	635	885	105.28 ± 3.47 ^a^
Tridecane	629-50-5	33.00	2.24	24.24	702	873	13.58 ± 0.20 ^b^	785	881	24.94 ± 0.92 ^b^	641	856	218.36 ± 4.57 ^a^
Tetradecane	629-59-4	37.10	2.26	33.93	ND	ND	ND	789	869	18.99 ± 0.19 ^b^	866	902	120.69 ± 2.73 ^a^
Pentadecane	629-62-9	40.90	2.32	40.39	741	876	36.87 ± 1.24 ^b^	820	899	19.19 ± 0.45 ^c^	812	918	149.45 ± 3.76 ^a^
Hexadecane	544-76-3	44.60	2.36	43.46	825	902	57.28 ± 2.10 ^b^	800	879	36.13 ± 1.74 ^c^	891	896	98.12 ± 2.75 ^a^
Heptadecane	629-78-7	48.10	2.40	26.66	656	828	13.69 ± 0.21 ^b^	837	880	25.22 ± 0.09 ^b^	841	869	422.99 ± 11.60 ^a^
Octodecane	593-45-3	51.90	1.90	3.85	847	903	118.93 ± 3.00 ^a^	706	811	13.22 ± 0.21 ^c^	728	867	105.75 ± 3.47 ^b^
Nonadecane	629-92-5	54.80	1.48	18.46	ND	ND	ND	719	846	25.04 ± 0.27 ^b^	803	884	185.11 ± 3.27 ^a^
Eicosane	112-95-8	58.00	1.54	38.71	818	873	27.36 ± 1.24 ^b^	764	866	14.21 ± 0.23 ^c^	731	866	49.03 ± 2.08 ^a^
Heneicosane	629-94-7	60.90	1.56	20.40	802	877	28.11 ± 1.59 ^b^	719	851	35.14 ± 1.32 ^b^	741	850	410.08 ± 15.08 ^a^
Tetracosane	646-31-1	63.20	2.60	12.71	ND	ND	ND	843	878	13.97 ± 0.36 ^b^	606	801	136.13 ± 3.27 ^a^
Total							362.52 ± 10.87 ^b^			329.43 ± 6.90 ^b^			2177.70 ± 59.06 ^a^
**Others**													
Methanethiol	74-93-1	4.10	2.70	93.20	689	890	33.35 ± 1.98 ^b^	471	895	12.88 ± 0.08 ^c^	475	893	104.15 ± 1.42 ^a^
Diallyl disulphide	2179-57-9	22.60	2.22	89.79	804	806	33.31 ± 0.77 ^a^	796	800	13.56 ± 0.58 ^b^	789	806	16.56 ± 0.71 ^b^
2-methyl-naphthalene	91-57-6	32.50	2.86	49-13	756	868	43.11 ± 0.77 ^a^	805	859	15.17 ± 0.80 ^b^	ND	ND	ND
Styrene	100-42-5	13.80	1.80	45.79	589	876	52.28 ± 0.94	ND	ND	ND	ND	ND	ND
Carbon disulfide	75-15-0	4.70	1.98	81.00	636	897	100.14 ± 1.16 ^a^	ND	ND	ND	710	894	54.52 ± 0.92 ^b^
Benzonitrile	100-47-0	17.60	3.10	70.51	820	888	31.90 ± 0.76 ^a^	810	903	13.22 ± 0.30 ^b^	ND	ND	ND
1,2-dimethoxy-benzene	91-16-7	25.50	3.02	46.54	611	816	122.57 ± 2.57 ^a^	ND	ND	ND	622	834	40.62 ± 0.81 ^b^
1,2,3-Trimethoxybenzene	634-34-6	32.80	3.24	86.87	681	859	74.49 ± 1.50	ND	ND	ND	ND	ND	ND
Indene	95-13-6	21.00	2.44	36.95	771	835	147.65 ± 2.90 ^a^	666	862	52.08 ± 1.52 ^b^	ND	ND	ND
Azulene	275-51-4	27.20	3.92	63.64	894	911	39.83 ± 1.86 ^a^	740	866	19.79 ± 0.66 ^b^	ND	ND	ND
Naphthalene	275-51-4	27.30	3.88	50.17	898	936	115.41 ± 2.41 ^a^	759	902	19.01 ± 0.24 ^b^	ND	ND	ND
Total							792.05 ± 17.62 ^a^			145.71 ± 4.17 ^c^			215.86 ± 3.86 ^b^

Different lowercase letters (^a–c^) in the same row indicate significant differences among different samples (*p* < 0.05). ND: not detected. DM: dry matter. TSS: sausages smoked with traditional smouldering smoke; ISS: sausages smoked with industrial smouldering smoke; ILS: sausages smoked with industrial liquid smoke.

## Data Availability

The data presented in this study are available in the article.
